# Young Children's Probability of Dying Before and After Their Mother's Death: A Rural South African Population-Based Surveillance Study

**DOI:** 10.1371/journal.pmed.1001409

**Published:** 2013-03-26

**Authors:** Samuel J. Clark, Kathleen Kahn, Brian Houle, Adriane Arteche, Mark A. Collinson, Stephen M. Tollman, Alan Stein

**Affiliations:** 1 Department of Sociology, University of Washington, Seattle, Washington, United States of America; 2 Institute of Behavioral Science, University of Colorado at Boulder, Boulder, Colorado, United States of America; 3 MRC/Wits Rural Public Health and Health Transitions Research Unit (Agincourt), School of Public Health, Faculty of Health Sciences, University of the Witwatersrand, Johannesburg, South Africa; 4 INDEPTH Network, Accra, Ghana; 5 Centre for Global Health Research, Umeå University, Umeå, Sweden; 6 The Section of Child and Adolescent Psychiatry, Department of Psychiatry, University of Oxford, Oxford, United Kingdom; Makerere University Medical School, Uganda

## Abstract

**Background:**

There is evidence that a young child's risk of dying increases following the mother's death, but little is known about the risk when the mother becomes very ill prior to her death. We hypothesized that children would be more likely to die during the period several months before their mother's death, as well as for several months after her death. Therefore we investigated the relationship between young children's likelihood of dying and the timing of their mother's death and, in particular, the existence of a critical period of increased risk.

**Methods and Findings:**

Data from a health and socio-demographic surveillance system in rural South Africa were collected on children 0–5 y of age from 1 January 1994 to 31 December 2008. Discrete time survival analysis was used to estimate children's probability of dying before and after their mother's death, accounting for moderators. 1,244 children (3% of sample) died from 1994 to 2008. The probability of child death began to rise 6–11 mo prior to the mother's death and increased markedly during the 2 mo immediately before the month of her death (odds ratio [OR] 7.1 [95% CI 3.9–12.7]), in the month of her death (OR 12.6 [6.2–25.3]), and during the 2 mo following her death (OR 7.0 [3.2–15.6]). This increase in the probability of dying was more pronounced for children whose mothers died of AIDS or tuberculosis compared to other causes of death, but the pattern remained for causes unrelated to AIDS/tuberculosis. Infants aged 0–6 mo at the time of their mother's death were nine times more likely to die than children aged 2–5 y. The limitations of the study included the lack of knowledge about precisely when a very ill mother will die, a lack of information about child nutrition and care, and the diagnosis of AIDS deaths by verbal autopsy rather than serostatus.

**Conclusions:**

Young children in lower income settings are more likely to die not only after their mother's death but also in the months before, when she is seriously ill. Interventions are urgently needed to support families both when the mother becomes very ill and after her death.

*Please see later in the article for the Editors' Summary*

## Abstract 2

Brian Houle and colleagues examine the temporal relationship between mother and child death by using 15 years of data (1994–2008) from household surveys conducted in the Agincourt sub-district of South Africa.

## Editors' Summary

**Background:**

Over the past few years, there has been enormous international effort to meet the target set by Millennium Development Goal 4—to reduce the under-five child mortality rate by two-thirds from the 1990 level by 2015. There has been some encouraging progress, and according to the latest figures from the World Health Organization, in 2011, just under 7 million children less than five years died, a fall of almost 3 million from a decade ago. However, such efforts must also consider the health of the mother, as it is now also well established that the health of children is intrinsically linked to their mother's health: there is strong evidence from low- and middle-income countries that children's risk of dying increases around the time of their mother's death, particularly relating to the HIV pandemic in Africa.

**Why Was This Study Done?:**

Previous studies examining the timing of a child's death relative to that of their mother have mainly focused on the period after the mother's death. So far, there have been few studies examining the link between a child's death and the period when his/her mother becomes ill and unable to care for and feed her child. In this study from the Agincourt sub-district in northeast South Africa, the researchers investigated the relationship between young children's chance (odds) of dying and the timing of their mother's death, particularly to examine whether there were critical periods of risk for children before their mother's death.

**What Did the Researchers Do and Find?:**

The researchers used the health and socio-demographic surveillance system in the area, which had 15 years (1994–2008) of information from yearly household surveys. The researchers focused on young children (0–6 months, 7–23 months, and 24–59 months) whose mothers had died, and through a statistical model, analysed the changes in the child's chance (odds) of dying from a year before the mother's death through to any time after her death during the study period. The cause of the mother's death was identified from verbal autopsy and categorized as being related to AIDS or tuberculosis (chronic) or other (mostly acute) causes not related to these infections. The researchers took other factors into account in their analysis and compared the odds of dying for children whose mothers died with those whose mothers were alive.

Using these methods, the researchers found that a total of 1,244 children (3% of the total sample) died between 1994 and 2008. Importantly, the researchers found that although the period when children are more likely to die began to increase in the period 6–11 months before their mother's death, there were three distinct periods of a much higher chance (odds) of death: the period 1–2 months before the month in which their mother died (odds ratio 7.1), the month of her death (odds ratio 12.6), and the period 1–2 months following her death (odds ratio 7.0). Furthermore, during the five-month period around the time of their mother's death, children (both boys and girls) aged 0–6 months were about nine times more likely to die than children aged 24–59 months. Finally, children were about 1.5 times more likely to die if their mother died of an AIDS/tuberculosis-related cause.

**What Do These Findings Mean?:**

These finding suggest that in low-income settings, young children are more likely to die in the months before their mother's death, when she is seriously ill, not just in the period after her death. The chance of dying is particularly increased in very young children (0–6 months) and in children whose mother died of HIV/tuberculosis-related causes. Although this study had several limitations, such as limited information on the child's cause of death, this study highlights the urgent need for proactive and coordinated community-based interventions to support families, especially vulnerable children, when a mother becomes seriously ill, in addition to the period following her death.

**Additional Information:**

Please access these websites via the online version of this summary at http://dx.doi.org/10.1371/journal.pmed.1001409.

- The Countdown to 2015 initiative has the latest country information on progress in reducing maternal, neonatal, and child deaths
- The World Health Organization has more information on Millennium Development Goal 4
- The Joint United Nations Joint Programme on HIV/AIDS has information about the number of deaths from HIV-related causes
- MRC/Wits Rural Public Health and Health Transitions Research Unit (Agincourt) has more information on the research platform that made this study possible

## Introduction

Maternal and child mortality are major challenges across the world, and reducing these are key millennium developmental goals. There is good evidence from low- and middle-income countries that children's risk of dying increases around the time of their mother's death [1],[2]. Over the past twenty years, the HIV pandemic in Africa has added to this already serious problem [2]–[8]. Critically, it is not only HIV-positive young children of HIV-positive mothers but also HIV-negative children of HIV-positive mothers who face a strikingly elevated risk of dying around the time of their mother's death. In sub-Saharan Africa, approximately 53% of HIV-positive and 8% of HIV-negative children die before their second birthday [2], although very recently the survival rates of HIV-positive children have improved with the rollout of antiretroviral medication [9],[10].

Studies examining the timing of a child's death have focused on the period following the mother's death [1],[11],[12]. For example, in a study in Bangladesh, Ronsmans and colleagues [1] found that many of the children who died did so within 2 mo of their mother's death, if the mother died during the child's first year of life. If the mother died later, the mean time to child death for those who died during the study was just over 9 mo.

The period when a mother becomes ill and unable to care for and feed her child, however, has remained almost entirely unexplored. An analysis of pooled data from three HIV cohort studies in East Africa did suggest that children's risk of dying may increase in the year before a mother dies [13]. However, this study was unable to isolate an effect for the period prior to the mother's death that was separate from the effect after her death. This study raised the important possibility of an effect in the period before the mother's death.

The temporal relationship between maternal and child death carries implications for policies and interventions to enhance child survival, particularly in populations with high levels of female adult mortality. If the period of illness shortly before a mother's death puts her children at high risk, programmes need to identify mothers at high risk of dying and intervene not only to avert the mother's death, but also to protect children who may be at increased risk due to maternal-illness-related maternal neglect, as well as to prepare to support the children in the event that the mother dies. Community-based interventions to help families in low- and middle-income countries, especially where HIV is endemic, are now recognised as critical in reducing maternal and child mortality [14]. Further evidence is urgently required to inform such interventions.

We aimed to investigate the relationship between young children's probability of dying and the timing of their mother's death, and in particular to examine whether there were critical periods of risk for children before their mother's death. Research took place in a health and socio-demographic surveillance system (HDSS) site covering the Agincourt sub-district in northeast South Africa, close to the South Africa–Mozambique border. Prior work in this population showed a marked rise in child mortality concurrent with the increase in HIV-related mortality in women of reproductive age [4]. Through annual household surveys over a period of 15 y, mother and child records have been carefully linked, which allows examination of the temporal relationship between mother and child death.

## Methods

The Agincourt HDSS was reviewed and approved by the Committee for Research on Human Subjects (Medical) of the University of the Witwatersrand (protocol M960720 and M081145). Informed consent is obtained at the individual and household level at each follow-up visit, while community consent from civic and traditional leadership was secured at the start of surveillance and is reaffirmed from time to time.

For young children whose mothers had died, we investigated changes in the probability of dying starting a year before the mother's death through to any time after her death during the study period. We controlled for a variety of covariates (see below) and made comparisons to children with surviving mothers. We also investigated the effect of child age on the relationship between the probability of dying and the timing of maternal death using developmentally important age groups: 0–6 mo, 7–23 mo, and 24–59 mo. We then examined the effect of the cause of the mother's death, specifically identifying (chronic) causes related to AIDS or tuberculosis (TB) and (largely acute) causes not related to AIDS/TB. Finally, we examined the effect of historical period on the relationships identified, accounting for the periods before and after the HIV epidemic produced a major effect on the study population.

### Sample and Data Acquisition

Data for this investigation describe the demographic history of the population living in the rural Agincourt sub-district of Bushbuckridge, Mpumalanga Province, South Africa. The population has hosted an HDSS since 1992. We used the data from 1 January 1994 to 31 December 2008, which contain records of approximately 82,000 people living in 21 villages. Trained fieldworkers interviewed the most knowledgeable person in each household, annually updating information on all vital events (births and deaths) and their timing, movements (in- and out-migration), and nuptial events. Household-level socio-economic indicators, a variety of health-related indicators (such as child morbidity and health-care utilisation), and an array of related information were collected less frequently but at regular intervals [15]. The statistical models presented in this study include only the variables identified in Tables 1 and 2, which in all cases are derived from information describing vital events collected during annual censuses between 1994 and 2008.

**Table 1:** Characteristics of children and their mothers for the periods 1994–1998 and 1999–2008, Agincourt sub-district, South Africa.

Population	Characteristic	Total Children (*n* = 41,584)	Children Whose Mother Died (*n* = 950)	Children Whose Mother Survived (*n* = 40,634)
**Children**	**Child Sex**			
	Male	20,886	509	20,377
	Female	20,698	441	20,257
	**Mean age: years (std. dev.)**	1.98 (1.42)	2.10 (1.44)	1.97 (1.42)
	**Mean age by period: years (std. dev.)**			
	1994–1998	2.03 (1.42)	2.08 (1.42)	2.03 (1.41)
	1999–2008	1.95 (1.42)	2.11 (1.45)	1.94 (1.42)
	**Mean age at death: months (std. dev.)**	13.46 (12.98)	13.76 (13.62)	13.41 (12.88)
	**Number of child deaths**	1,244	180	1,064
	**AIDS/TB-related child deaths**	216	62	154
	**Observed child-months before/after mother's death [mortality rate per 1,000]**			
	Alive or 12+ mo before—reference			1,537,831 [0.9]
	6 to 11 mo before		1,552 [12.9]	
	3 to 5 mo before		740 [13.5]	
	1 to 2 mo before		462 [41.1]	
	Month of mother's death		193 [72.5]	
	1 to 2 mo after		295 [30.5]	
	3 to 5 mo after		368 [13.6]	
	6+ mo after		1,677 [3.0]	
	**Child deaths by period**			
	1994–1998	265	30	235
	1999–2008	979	150	829
**Mothers**	**Mean age: years (std. dev.)**	28.82 (7.81)	34.83 (8.63)^a^	
	**Mean age by period: years (std. dev.)**			
	1994–1998	29.24 (8.04)		
	1999–2008	28.59 (7.66)		
	**Number of deaths**		950	
	**Mother deaths by period**			
	1994–1998		89	
	1999–2008		861	
	**AIDS/TB-related deaths**		259	

a For those mothers who died, mean age represents the average age at time of death. There were a total of 13,060 households and 24,860 mothers.

std. dev., standard deviation.

**Table 2:** Results of discrete time survival analysis of child death: odds ratios from multi-level logistic regression of monthly child deaths on months mother dead and controls, Agincourt, South Africa 1994–2008.

Variable	OR or Parameter	95% CI	*p*-Value
**Months before/after mother's death**			
Alive or 12+ mo before—reference	1.000	—	—
6 to 11 mo before	**1.947**	**(1.127, 3.363)**	**0.017**
3 to 5 mo before	**2.109**	**(1.033, 4.306)**	**0.041**
1 to 2 mo before	**7.053**	**(3.923, 12.680)**	**<0.001**
Month of mother's death	**12.554**	**(6.219, 25.344)**	**<0.001**
1 to 2 mo after	**7.009**	**(3.157, 15.560)**	**<0.001**
3 to 5 mo after	**4.025**	**(1.532, 10.577)**	**0.005**
6+ mo after	1.591	(0.610, 4.145)	0.342
**Child sex**			
Female—reference	1.000	—	—
Male	1.084	(0.963, 1.221)	0.182
**Child age (months)**			
0–6—reference	1.000	—	—
7–23	**0.47**	**(0.413, 0.533)**	**<0.001**
24–59	**0.107**	**(0.091, 0.125)**	**<0.001**
**Time period**			
1994–1998—reference	1.000	—	—
1999–2008	**1.956**	**(1.683, 2.273)**	**<0.001**
**Mother's cause of death**			
Alive or no cause—reference	1.000	—	—
All causes except AIDS/TB and accidents	**3.933**	**(2.301, 6.720)**	**<0.001**
AIDS/TB	**6.605**	**(3.423, 12.748)**	**<0.001**
**Multiple birth**			
Singleton—reference	1.000	—	—
Multiple	**2.073**	**(1.571, 2.736)**	**<0.001**
**Interactions between time period and mother's cause of death**			
1999–2008 × all causes of mother's death except AIDS/TB	**2.140**	**(1.191, 3.846)**	**0.011**
1999–2008 × AIDS/TB-related cause of mother's death	**2.242**	**(1.064, 4.726)**	**0.034**
**σ^2^_household_**	0.205	(0.051, 0.821)	—
**σ^2^_mother_**	1.120	(0.745, 1.685)	—

Multi-level logistic regression of child death on months before/after mother's death, sex, time period, multiple birth, and mother's cause of death. Unit of analysis is “child-month”. Explanatory variables defined at beginning of each month; child death can occur at any time within the month. Bold indicates statistically significant at the 5% level.

Cause of death information was obtained from verbal autopsy. Specially trained lay fieldworkers conducted verbal autopsies on every death recorded during a census update round. The closest caregiver of the deceased person was interviewed using a standardised instrument. The interview was in the respondent's primary language (XiTsonga) and included culturally sensitive questions on signs and symptoms during the terminal illness, lifestyle risk factors, and treatment sought. Two medical practitioners reviewed the responses independently and determined the probable underlying, immediate, and contributory causes of death. If their findings conflicted, a third practitioner (blind to the other physicians' diagnoses) assigned a cause. If this third diagnosis agreed with one of the other two, it was accepted as the probable cause of death; if not, the death was categorised as “ill-defined”. Causes were coded using the International Classification of Diseases (ICD-10). The verbal autopsy process [16] was validated in the mid-1990s [17] and again in 2005 for HIV-related deaths. Misclassification with co-morbid conditions, particularly TB, reduced the sensitivity for both AIDS and TB diagnoses. These conditions were therefore combined into a single AIDS/TB category with sensitivity and specificity of nearly 80%, an approach used elsewhere [18],[19].

Data collected was split into two time periods: 1994–1998 and 1999–2008. The split between 1998 and 1999 marked the end of the rise in HIV prevalence in the study site, which was followed by a plateau at a high level for an extended period thereafter [20]. It is therefore a reasonable breakpoint that defines periods prior to and after HIV had a major effect on mortality in the area. There was no other major change affecting the study population over the time period of the analysis.

### Statistical Analysis

We modelled child mortality using discrete time survival analysis [21]. This modelling approach is similar to the Cox proportional hazards approach (i.e., time-to-event survival analysis) but has important advantages. Discrete time survival analysis does not require an assumption of proportional hazards and allows greater flexibility in modelling time-varying covariates and their interactions. Further, the approach produces reasonable predicted probabilities that can be interpreted independently and hence is not affected by an inability to estimate a reliable base hazard (often the case with Cox proportional hazards models). As our main aim was to examine the change in risk as a function of timing, controlling for historical period and child age, we preferred the discrete time survival analysis approach, which allowed us to easily and transparently specify these complex temporal relationships without requiring proportionality of risks. The resulting predicted probabilities are easy to interpret and can be used directly in traditional mortality analysis methods, such as life tables. In addition to the discrete time analysis, we fit the equivalent Cox proportional hazards model, and the results are effectively identical.

Data were organised as child-months, where each child was at risk during each month observed from birth up to and including death or censoring (i.e., exit from the population). Thus, there was one child-month for each observed person-month lived by a child in the study area. A child who died at 12 mo of age and was within the study population from birth would contribute 7 mo in the 0–6 mo age category and 5 mo in the 7–23 mo age category, up to and including the month of death and updating relevant covariates at the beginning of each month. We used multi-level logistic regression with random mother and household intercepts to estimate the monthly probability of dying conditional on the state of the child at the beginning of the month. A strength of this method is that it accounts for unobserved heterogeneity between mothers and households. In other words, the method accounts for shared aspects of mothers and households that are experienced by all children of a particular mother in a specific household.

We assessed the relative importance of time-invariant and time-changing explanatory variables, with values defined at the beginning of each child-month (i.e., the probability of death conditioned on the values of covariates at the beginning of the month). These included sex, age, time before/after mother's death, multiple birth, historical time period, and maternal cause of death. Maternal cause of death was categorised as death due to AIDS/TB or death from all other causes except “accidental” deaths (*n* = 101 child-months). Accidental deaths were excluded, as their causes and context tend to differ from other causes of maternal death. Similarly, stillbirths were excluded. Time periods relative to maternal death were defined based on the number of months up to and following a mother's death (12+ before, 6–11 mo before, 3–5 mo before, 1–2 mo before, month of mother's death, 1–2 mo after, 3–5 mo after, 6+ mo after).

A model including household socio-economic status (SES), based on a household asset index, was fit to determine whether variation in SES explained our main finding. While household SES does affect the risk of dying, this effect is independent of that associated with timing of maternal death. We do not present these results because household SES was available only after 2001. This shortened the historical scope of the dataset to include only time when HIV prevalence was high, and more importantly, reduced the sample size by two-thirds, which dramatically diminished the statistical power of the model that included SES. All models were fit using the statistical package Stata [22].

## Results

Demographic characteristics are shown in Table 1. A total of 1,244 children (3% of the sample) died between 1994 and 2008. A full model was fit including sex, age, time before/after mother's death, multiple birth, historical time period, and maternal cause of death. An interaction between cause of maternal death and historical time period significantly improved model fit (*p* = 0.021). A multi-level model including random intercepts for the mother and household improved model fit according to the Bayesian Information Criterion [23] and resulted in the final model (see Table 2).

**Figure 1:**
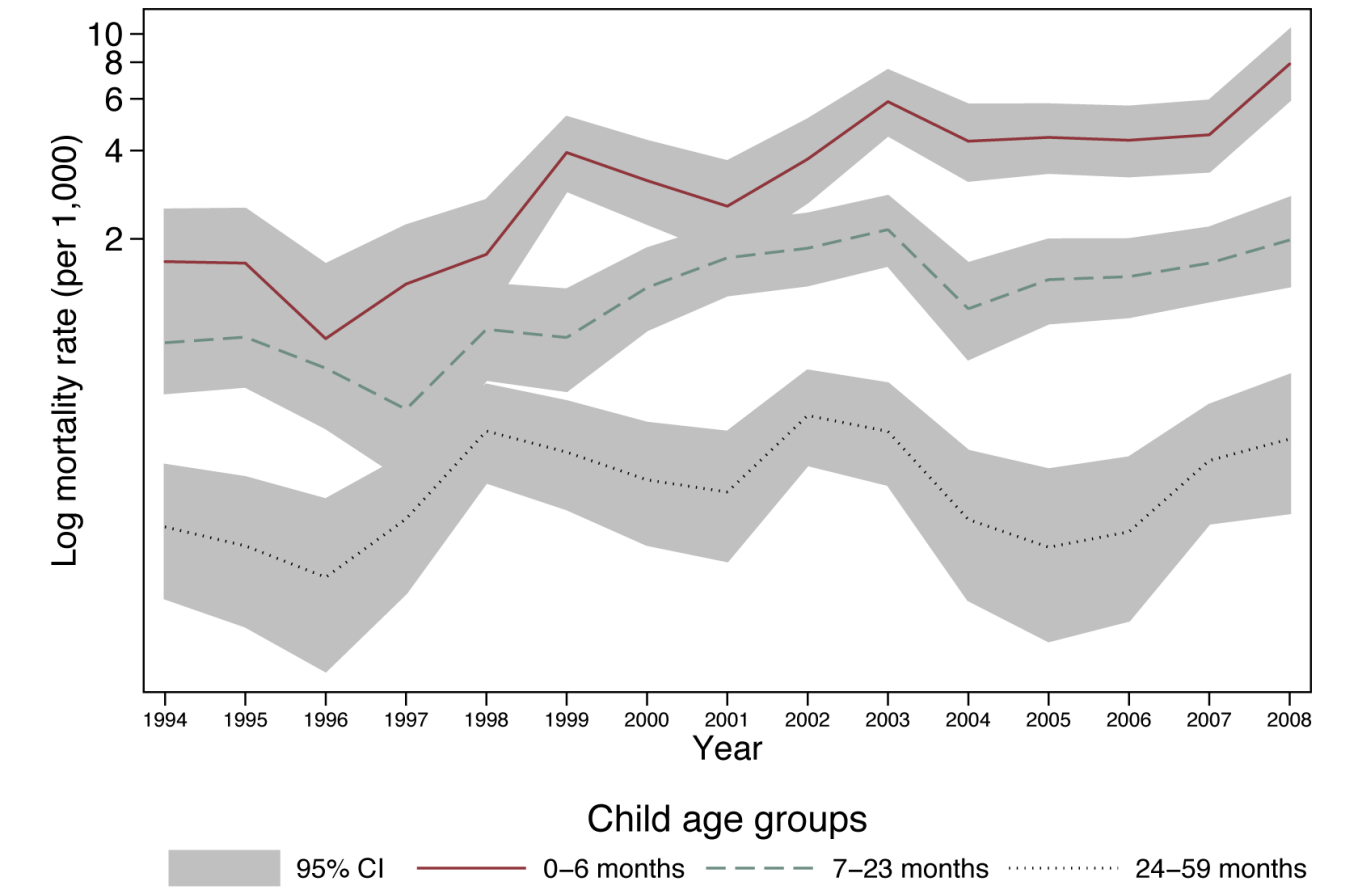
Child mortality rate in Agincourt sub-district, South Africa (1994–2008) by year and child age. Shading indicates Poisson 95% confidence intervals.

Estimation results from the full model are presented in Table 2. This table contains odds ratios (ORs) (roughly analogous to the risk ratios from a Cox proportional hazards model) resulting from the multi-level logistic regression of child death on the explanatory variables. A child's monthly probability of dying is high immediately after birth through 6 mo of age and decreases with age. Contrary to trends in much of the world [24]–[26], in this population children's probability of dying has increased over the past decade (Figure 1), with marked increases in the probability of dying during the period 1999–2008, when HIV/AIDS became highly prevalent (Table 2). Being part of a multiple birth also increased the probability of dying compared to a singleton birth.

### Timing of Mother and Child Deaths

**Figure 2:**
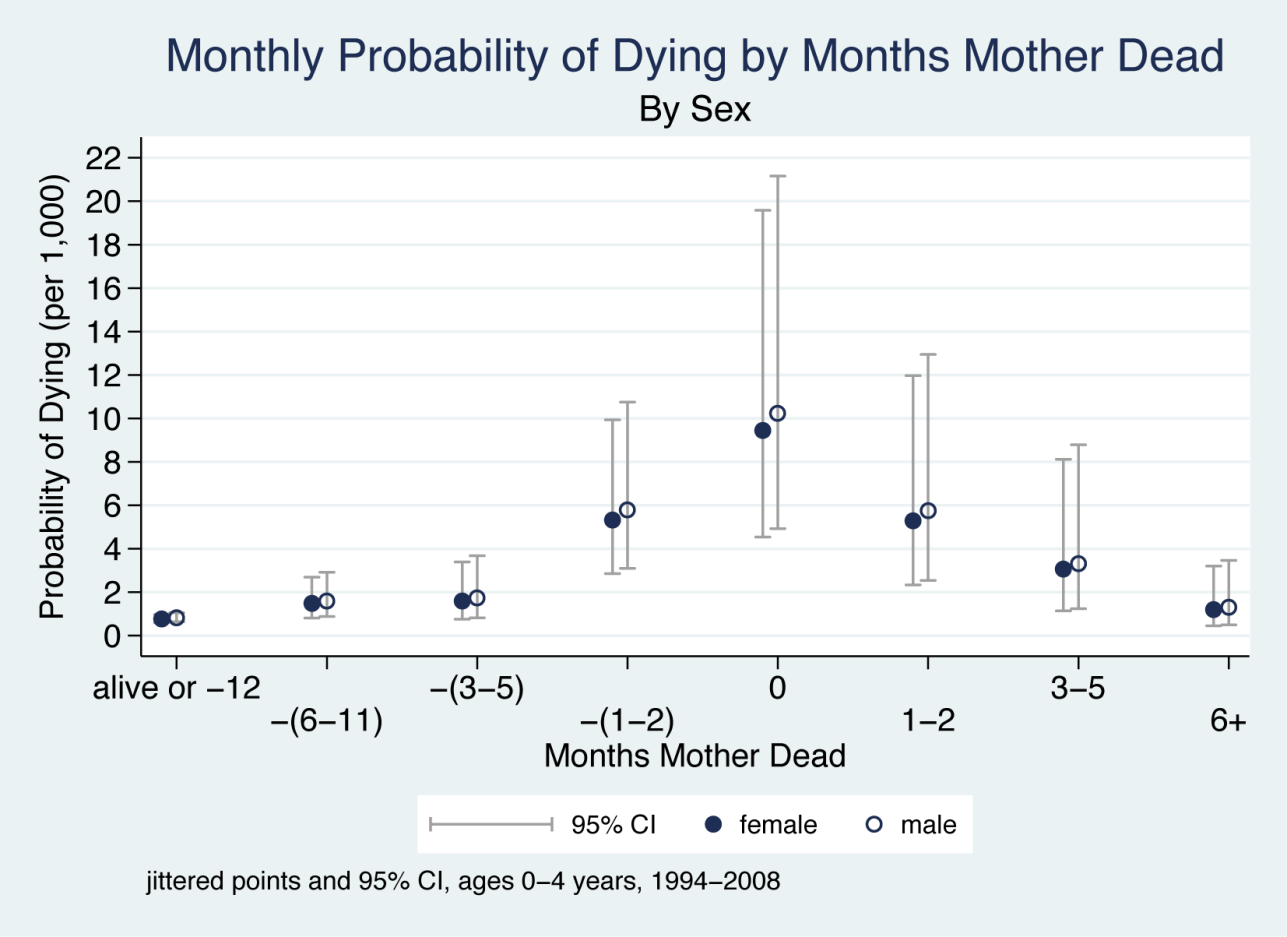
Monthly probability of child death in Agincourt sub-district, South Africa (1994–2008) by time before/after mother's death and sex of child. On the *x-*axis, the mother's death occurs in month 0, and time periods before and after her death are shown to the left and right of month 0, respectively. Children whose mother did not die during the study are included in the time period “alive or −12”. Jittered (offset) points to reduce overplotting.

The ORs relating to months before/after mother's death in Table 2 describe changes in a child's odds of dying as a function of months before and after the mother's death, adjusting for sex, age, multiple birth, time period, and mother's cause of death, with the reference group being children whose mothers survived or died 1 y or more into the future. The monthly probabilities of dying associated with these ORs are presented in Figure 2.

The main findings are that the probability of a child dying begins to increase in the period 6–11 mo before the mother's death, is very high within the 2 mo before the month of her death, peaks in the month of her death, is high for the 2 mo following her death, and then decreases six or more months after her death. There are thus three periods of highly elevated probability of dying: the period 1–2 mo before the death of the mother (OR 7.1), the month of her death (OR 12.6), and the period 1–2 mo following her death (OR 7.0). Thus, the probability of the child dying both before and after the mother's death is dramatically elevated.

### Child's Age

**Figure 3:**
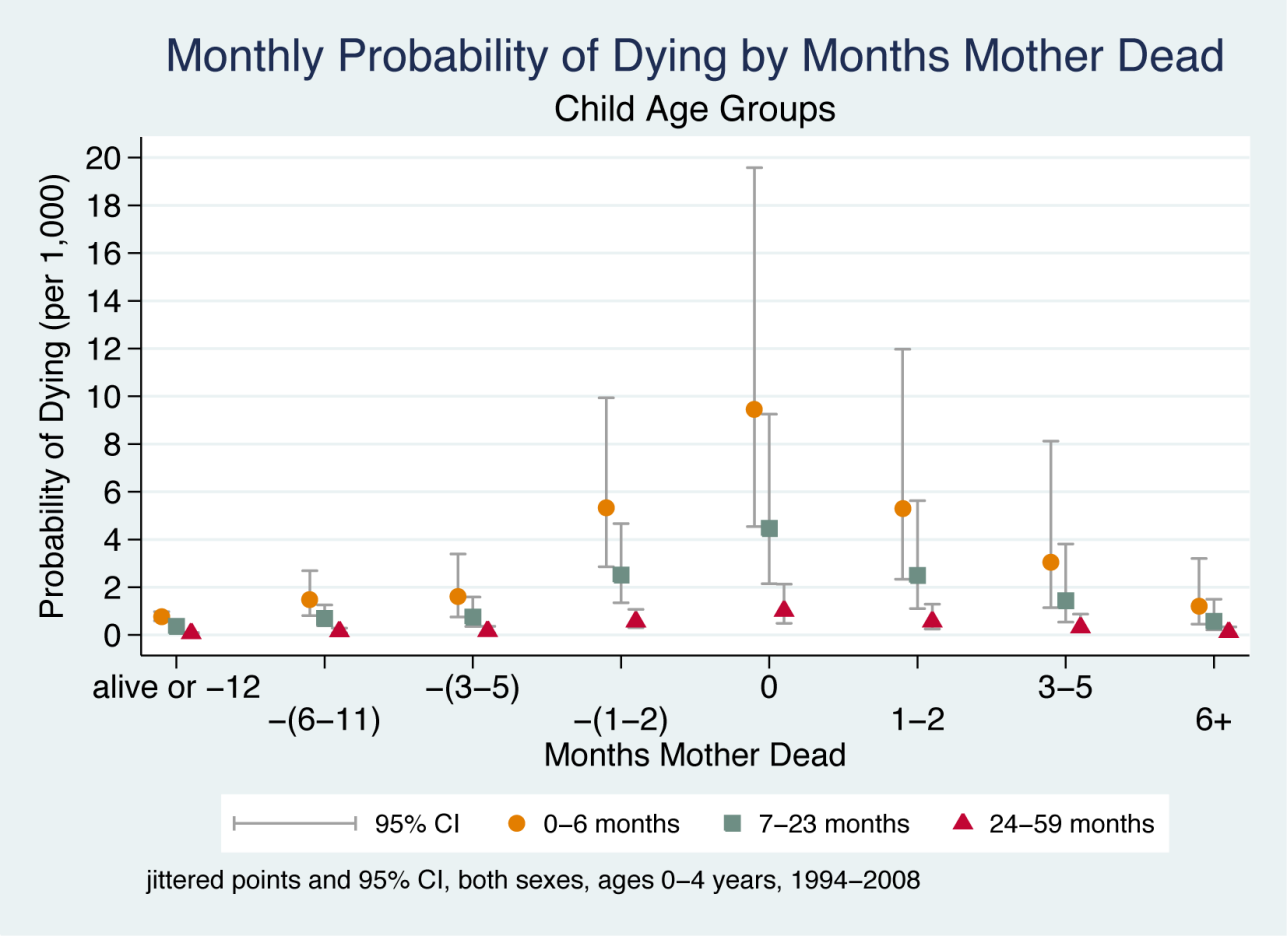
Monthly probability of child death in Agincourt sub-district, South Africa (1994–2008) by time before/after mother's death and child age. On the *x-*axis, the mother's death occurs in month 0, and time periods before and after her death are shown to the left and right of month 0, respectively. Children whose mother did not die during the study are included in the time period “alive or −12”. Jittered (offset) points to reduce overplotting.

Figure 3 shows a child's probability of dying in the time periods before and after their mother's death, taking account of the child's age. These child age effects are independent of other explanatory variables in the model (i.e., interactions of age with other variables do not improve overall model fit). Hence, child age affects the probability of dying in unchanging ratios whether or not there is a maternal death. However, when the much higher odds of dying experienced by infants 0–6 mo are multiplied by the ORs associated with the 5-mo period around a maternal death, it is clear that very young children have a far greater probability of dying compared to older children. During the 5-mo period around the time of maternal death, children aged 0–6 mo are approximately nine times more likely to die than children aged 24–59 mo. This finding applies equally to boys and girls.

### Maternal Cause of Death

**Figure 4:**
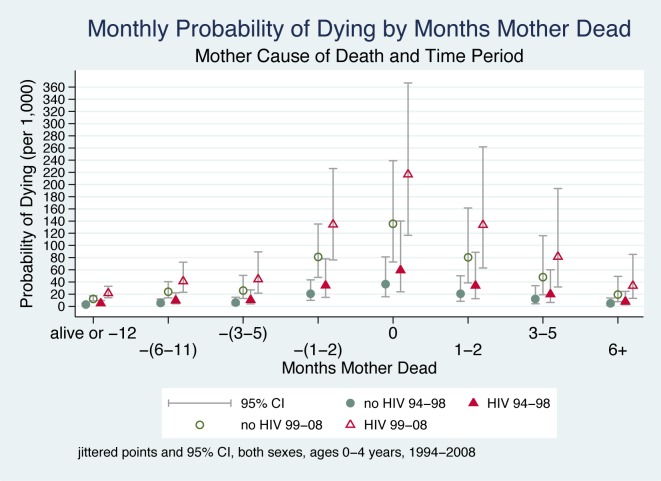
Monthly probability of child death in Agincourt sub-district, South Africa (1994–2008) by time before/after mother's death, time period, and cause of mother's death. The time periods (1994–1998 and 1999–2008) were selected to fall either side of a historical breakpoint separating the periods before and after HIV produced a major effect on mortality. On the *x-*axis, the mother's death occurs in month 0, and time periods before and after her death are shown to the left and right of month 0, respectively. Children whose mother did not die during the study are included in the time period “alive or −12”. Jittered (offset) points to reduce overplotting.

Maternal cause of death was found to have an important effect on a child's probability of dying, and the data demonstrate that this effect is sensitive to the historical time period of the mother's death (1994–1998 versus 1999–2008). Figure 4 shows a child's probability of dying in the time periods before and after the mother's death according to her cause of death (classified as non-AIDS/TB-related and AIDS/TB-related) as well as the historical period (an interaction between mother's cause of death and the timing of death did not improve model fit). The odds of dying for a child were about 1.5 times greater if the mother died of an AIDS/TB-related cause than if she died of other causes. Further, as would be expected during an accelerating HIV epidemic, there was an important and statistically significant interaction between mother's cause of death and the historical time period (Table 2). This interaction effect raised the odds in the later period by about 5%. Figure 4 shows the greater odds of dying across both time periods if the mother died of an AIDS/TB-related cause and, further, that this effect was far larger in the later period (1999–2008), by which time the HIV epidemic was already having an extensive public health impact through higher mortality of both mothers and infants.

## Discussion

We found that the critical period of elevated odds of dying for children began several months before their mother died and continued for several months after her death, with extreme vulnerability in the 2 mo on either side of the month of her death. The odds of dying were far greater for infants 0–6 mo and remained high until 2 y of age, diminishing substantially after the child's second birthday. During the later period of analysis (1999–2008), when HIV prevalence was much higher, many more children and mothers died of AIDS/TB-related causes than in the earlier period (1994–1998). Our results indicate that all the effects observed were worse for children whose mothers died of AIDS/TB. Nonetheless, the pattern remained when the mother died of other causes.

Prospective data describing the interrelated life histories of mothers and children over a continuous 15-y period in an under-resourced setting allowed us to examine precisely the odds associated with different timings of mother and child death. Data collection covered important time periods during the evolution of the HIV epidemic in rural South Africa, permitting us to assess the effects of the changing HIV prevalence. Several interrelated factors provide possible explanations for our findings. Although we have no specific evidence that this was indeed the case in our study, nutrition and caregiving are the most likely major underlying causes of child mortality in the context of severe maternal illness and maternal death. Illness and death of a parent will affect any child deeply, but especially infants and preschool children who are entirely dependent on their primary caregivers [27]. Studies have demonstrated comparatively poor health among young children with caregivers unwell with HIV/AIDS [28],[29], and under-nutrition has been identified as the key underlying cause of children's deaths, especially in relation to infectious disease [30]. Thus, when a mother becomes very ill and is unable to care for and feed her child, whether by breastfeeding or providing substitute or complementary feeding, the risks to the child rise substantially. It is probable that child feeding is compromised by maternal illness during the critical periods identified, leading to poor nutrition and increased exposure and susceptibility to infection. Markedly lower survival among the youngest children (0–6 mo) is partly explained by their increased vulnerability. Following the death of the mother, part of the increased risk may be due to lower quality alternative feeding and care by adults from either the same household or others in an already resource-limited environment [31].

A major consequence of adult HIV/AIDS for families in poor and rural settings is impoverishment. Chronic illness and death consume a sizeable share of family resources and compromise a household's ability to care for other dependent members [32]. Prolonged adult illness and later death may lead to loss of a breadwinner, resulting in lowered household income [33]–[35] and reduced capacity to diversify livelihood strategies [33]. Available resources may be redirected to finance health care (including traditional healing) and funeral costs, leading to compromised nutrition in children through decreased food expenditure. Finally, although we did not investigate the contribution of other family members in the care of children during maternal illness, a potential added pressure is the increase in the number of dependents when affected children or vulnerable family members are cared for by other family members. Conversely, poverty is known to have marked adverse effects on parenting, causing stress, exhaustion, and absences to seek work [36],[37].

This investigation is limited in several ways. We did not have data on child nutrition, including breastfeeding or complementary feeding, in the context of maternal illness and death, so our discussion is based on the available literature. Further research is urgently needed to understand the mechanisms—malnutrition and others—linking maternal illness and death to increases in the risk of dying for children. This evidence is crucial to designing effective interventions. The absence of routine HIV serostatus testing prevents us from knowing the HIV status of individuals, and this limits our ability to investigate the direct role of HIV. Instead, we use a validated verbal autopsy to determine AIDS/TB-related and other causes of maternal death; however, for children there were too many deaths assigned as “undetermined” to draw conclusions. Annual retrospective data collection of deaths raises the potential for missing vital events. Recognising this, careful quality control procedures are in place, and it is unlikely that a mother's death would be missed. In addition to routine, detailed questioning during the annual census update, if a household member who was previously in the household is absent, an extensive follow-up is triggered to determine the fate of the missing person. It is possible that a birth followed by death less than a year later (between annual censuses) could be missed. To limit this problem, information on pregnancy status was routinely collected during each census round, with fieldworkers trained to carefully probe for pregnancy outcomes in the next round. Information on fathers and household SES does not cover the full analysis period, precluding us from examining the effects of paternal survival or poverty. Factors that increase mortality in mothers may also increase mortality in their children. We used multi-level modelling of mother and household dependence to take account of this point. It should also be noted that the relatively small sample sizes in some sub-groups resulted in wide 95% confidence intervals, which meant that some of our estimates did not have high precision. Furthermore, the period of our analysis is limited by the available data, which extends through 2008.

It is important to point out that it is not possible to know when a very seriously ill mother will die or if she will die. Although there is some evidence that maternal illness has important effects on children (for example [38]–[40]), more research is urgently required to understand more precisely when in the course of a mother's illness a child's life becomes in danger. In the meanwhile, when a mother is affected by a serious illness, to the extent that she is unable to care for and feed her young children and alternative care is not available, it is essential to intervene to ensure that adequate arrangements for continuing care are in place. Finally, our data do not include descriptions of illness and disability. As a result our analysis does not include instances of serious, debilitating maternal illness that did not result in death, and consequently, it is likely that we did not identify all of the child deaths associated with serious maternal illness. However, if anything, this point is likely to make the estimate of the child's probability of dying when the mother is gravely ill conservative.

Effective, affordable interventions appropriate for national delivery systems are urgently needed [30]. With the increasing recognition of the central importance of community- and home-based care programmes, strategies to identify and support vulnerable children and their families are a priority. Severe maternal illness coupled with declining ability to feed infants and children is a certain indicator of impending catastrophe, warranting immediate intervention. In many areas services and support are activated only once children become “orphaned” [41]. There is now some emerging evidence of the usefulness of palliative care in sub-Saharan Africa [42],[43]. Resting on community-based treatment programmes [14] that integrate health and social services, such care could respond to the needs of these families and provide training in basic care and nutrition. Increasing evidence suggests that community health workers as well as informal home-based carers could provide such care and support to families in these difficult circumstances [41]. The extensive report by the Joint Learning Initiative on Children and HIV/AIDS provides several examples of community-based interventions that have worked for children affected by HIV/AIDS in sub-Saharan Africa and Asia [41]. These examples could potentially be used when a mother is gravely ill. Hence, in many regions, there is the potential to develop sustainable interventions relatively quickly.

Strengthening health-care systems is vital and should include widespread screening for HIV and TB as well as timely treatment. Earlier treatment of these conditions should inevitably reduce adult and child mortality. Integrating maternal and child care within the primary health-care system, drawing on widely used access routes such as post-delivery assessments and immunisation services, is key to achieving this. Task shifting, a strategy to address personnel shortages by assigning activities to less skilled health workers [44], has been shown to maintain or improve quality of care in the management of routine childhood illness [45]. While the impact of task shifting in maternal care is more mixed, there is growing experience of effective delegation of responsibilities [46]. Task shifiting is an important potential avenue along which to intervene, but the evidence that this will reduce child mortality in the context of severe maternal illness or death remains to be demonstrated.

The increasing rollout of antiretroviral therapy to mothers with HIV should lead to improved child survival, and there is evidence for this in other southern African settings [47]. Future research will be required to examine whether these changes over time affect the relationship between mother and child death.

Pro-poor social policies can be important in supporting family and community efforts to protect vulnerable children. For example, children in rural South Africa benefit from the non-contributory old age pension, with evidence suggesting that households with a female pensioner miss fewer meals and have higher school enrolment [48],[49]. A child support grant from the South African Social Security Agency can be accessed by a primary caregiver for up to six non-biological children, making this a critical source of household income after the death of the mother [50].

These findings are from a defined geographic region on the border between South Africa and Mozambique and will need replication in other populations to strengthen the generalisability of the findings. Nonetheless, a number of features of the study setting are common to southern and eastern Africa, where many women have limited access to health care, and available health care is often of poor quality. The HIV infection rate in the study population was very similar to that of many other high prevalence areas of southern Africa [51]. Further, across much of the continent, there was a relatively late response to the HIV epidemic, with little coordinated response to help children whose mothers were terminally ill.

In conclusion, young children's survival is put at substantial risk when their mothers become very ill. In particular, a period of very high risk for the child occurs in the 2 mo prior to the month in which a mother dies and extends for 2 mo after. This effect is considerably greater when a mother has HIV/AIDS or TB, but the pattern is maintained for non-AIDS/TB-related causes. Proactive and coordinated community-based interventions are urgently needed to support families when a mother becomes seriously ill, as well as following her death.

## Abbreviations

HDSS: health and socio-demographic surveillance system
OR: odds ratio
SES: socio-economic status
TB: tuberculosis
